# Are complicated monochorionic twins more susceptible to indomethacin-induced fetal ductal constriction? Two cases of laser surgery for Twin-Twin Transfusion syndrome

**DOI:** 10.4274/tjod.78095

**Published:** 2018-09-03

**Authors:** Hakan Erenel, Mehmet Fatih Karslı, Sevim Özge Korkmaz, Cihat Şen

**Affiliations:** 1 İstanbul University Cerrahpaşa Faculty of Medicine, Department of Obstetrics and Gynecology, Division of Perinatal Medicine, İstanbul, Turkey

**Keywords:** Constriction, ductus arteriosus, indomethacin, preterm labor, Twin-Twin Transfusion syndrome

## Abstract

Indomethacin is a commonly used medication against preterm delivery. Several reports of fetal ductal constriction have been described after indomethacin use in the literature; however, there are no previously documented reports describing an association between Twin-Twin Transfusion syndrome and a constrictor effect of indomethacin on the ductus arteriosus. Two patients were referred to our department for Twin-Twin Transfusion syndrome and each underwent placental laser surgery. Constriction of the ductus arteriosus occurred as early as 20 and 24 weeks’ gestation following maternal use of indomethacin after laser surgery. Spontaneous amelioration was observed after discontinuation of the drug. The constrictor effect of indomethacin on the ductus arteriosus can be observed even after a single dose and as early as 20 weeks of gestation in complicated monochorionic twin pregnancies. We emphasize meticulous use of indomethacin in complicated monochorionic twin pregnancies because the constrictive effect seems to be independent of gestational age.

## Introduction

Monochorionic (MC) twins are a subtype of monozygotic (MZ) pregnancies and are seen in 0.3% of all pregnancies. MC diamniotic pregnancies are the most common subtype of MZ pregnancies and account for 70-75% of all MZ pregnancies^(1)^. Twin-Twin Transfusion syndrome (TTTS) is a complication of MC twin pregnancies and foetoscopic laser photocoagulation is one of the treatment options; however, laser surgery increases the risk of abortion/preterm delivery. Indomethacin is a commonly used medication against preterm delivery. Several reports of fetal ductal constriction have been described after indomethacin use in the literature. Here, we present two cases of antenatally diagnosed foetal ductal constriction in the donor co-twin after indomethacin use.

## Case Reports

### Case 1

**Figure 1:**
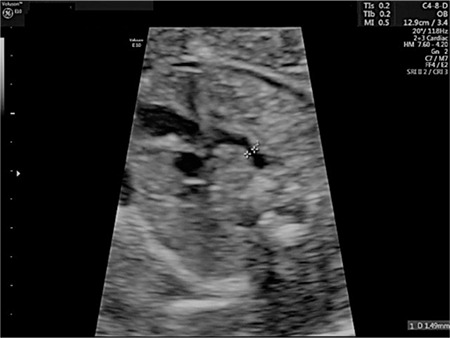
Narrowing in the ductus arteriosus (Case 1)

**Figure 2:**
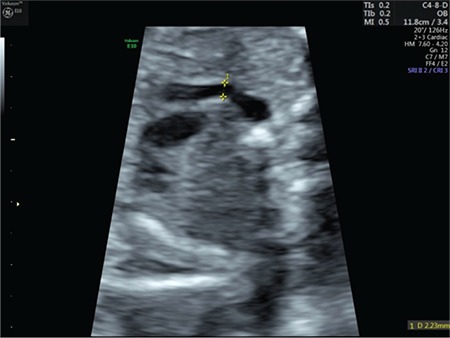
Transverse diameter of ductus arteriosus after resolution of the constriction (Case 1)

A 24-year-old gravida-3-para-2 at 24 weeks’ gestation was referred to our department for TTTS. Her physical and gynecologic examinations were usual. Fetal examination revealed a diamniotic MC twin pregnancy. The recipient twin was diagnosed as having polyhydramnios and the donor twin had oligohydramnios. The diagnosis of stage 3 TTTS was made on the basis of Quintero Staging system. A negative ductus venosus A-wave was observed in the recipient twin. The deepest vertical pocket was 140 mm for the recipient and 5 mm for the donor twin. The complications and prognosis of TTTS and risks of placental laser surgery were discussed with the family and the patient opted for placental laser surgery. The patient underwent placental laser surgery under local anaesthesia in operating room. On the day of the operation, 100 mg indomethacin was administered due to regular uterine contractions as rectal suppository once a day. Indomethacin administration was continued until postoperative day 2 and the contractions disappeared. On postoperative day 3, an ultrasound examination revealed pleural effusion, ascites, increased nuchal thickness, oligohydramnios, tricuspid regurgitation, and negative ductus venosus A-wave in the donor twin and normal Doppler findings in the recipient twin. A detailed examination of the ductus arteriosus showed ductal narrowing with a transverse diameter of 1.49 mm (<5^th^ percentile) (Figure 1)^(2)^. There was no turbulent flow or aliasing in the ductus arteriosus region and systolic and diastolic velocities were 69 and 6 cm/s, respectively. Although Doppler criteria of ductal constriction were not observed, hydrops was prominent. The indomethacin treatment was stopped. The direction of ductus venosus flow turned to positive on the next day and further examination revealed a 50% increase in the transverse diameter of the ductus arteriosus, and the ductal arch returned to its normal shape (Figure 2). The tricuspid regurgitation and ascites, and pleural effusion disappeared completely on postoperative days 5 and day 7, respectively. The patient was discharged on tenth day postoperatively. Her antenatal visits were uneventful until delivery. The patient underwent a cesarean section for breech-vertex presentation at 35 weeks’ gestation. Male infants weighing 2200 and 2100 g were delivered.

### Case 2

**Figure 3:**
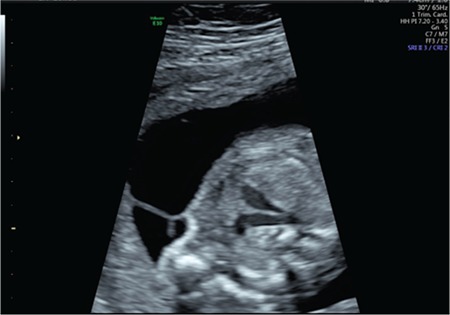
Narrowing in the ductus arteriosus (Case 2)

**Figure 4:**
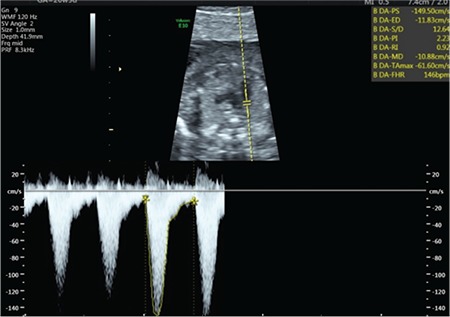
Elevated peak systolic velocity in the ductus arteriosus (Case 2)

**Figure 5:**
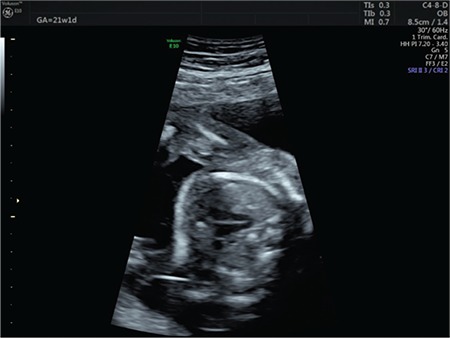
Transverse diameter of the ductus arteriosus after resolution of the constriction (Case 2)

A 25-year-old gravida-2-para-1 at 20 weeks’ gestation was referred to our department for TTTs. The patient reported abdominal bloating. The diagnosis of TTTs stage 2 was made on the basis of Quintero staging. The deepest vertical pocket was 154 mm for the recipient and 10 mm for the donor twin. The estimated foetal weight (EFW) was less than 10^th^ centile for donor twin. Placental laser surgery was performed after preoperative counselling. Postoperatively, a single-dose 100 mg indomethacin suppository was inserted into the rectum to prevent uterine contractions. On the next day (postoperative day 1), constriction of the ductus arteriosus and tricuspid regurgitation were observed in the donor twin. There was a marked ductal narrowing (Figure 3). The peak systolic velocity was 149 cm/s (Figure 4). On postoperative day 3, the transverse diameter of the ductus arteriosus and peak systolic velocity returned to normal (Figure 5). On postoperative day 5, the tricuspid regurgitation had disappeared and the patient was discharged. On the next day, the patient was admitted to the hospital due to regular contractions and cervical opening and she delivered at 21 weeks and 3 days’ gestation.

## Discussion

Preterm delivery is a major cause of perinatal morbidity and mortality. Indomethacin is relatively old and the most commonly used prostaglandin synthetase inhibitor, it has been used in preterm delivery since the 1970s. Maternal use of indomethacin is the most common cause of foetal ductus arteriosus constriction; however, idiopathic constriction of the fetal ductus arteriosus has also been described in the literature^(3)^. The diagnostic criteria of ductal constriction are as follows: a) presence of turbulent flow with a continuous pattern in the ductus arteriosus region (systolic and diastolic) in colour Doppler; b) systolic velocity ≥1.4 m/s; and c) diastolic velocity ≥0.3 m/s^(4)^. To the best of our knowledge, this is the first report of prenatal diagnosis of constriction of ductus arteriosus in as early as 20 and 24 weeks’ gestation following maternal use of indomethacin in complicated MC twins after laser surgery. Prostaglandin inhibitors such as indomethacin are tocolytic agents, which act by decreasing prostaglandin activity in the uterine myometrium. However, decreased prostaglandin synthesis in the foetal circulation may have adverse effects on the ductus arteriosus because prostaglandins mainstay ductal patency. Fetal ductal constriction is a distinct entity caused by different drugs (non-steroidal anti-inflammatory drugs, fluoxetine or abuse of some drugs); however, idiopathic/spontaneous constriction of foetal ductus arteriosus has also been described in the literature^(5)^.Tricuspid regurgitation, right ventricular dilatation, oligohydramnios, ascites, hydrops, and increased nuchal translucency are common foetal findings in such cases. A case series reported 45 cases of fetal ductus arteriosus constriction and closure, including 8 idiopathic forms. The most common finding of ductal constriction was tricuspid regurgitation, present in 86.6% of patients^(5)^. Although indomethacin was administered as a single dose at 20 weeks’ gestation and once a day for 2 days at 24 week’s gestation, our cases presented with constriction of the ductus arteriosus. As we know from previous reports, constriction of the ductus arteriosus is not dependent on serum indomethacin levels^(6)^. Vermillion et al.^(7) ^analysed the effect of indomethacin tocolysis on fetal ductus arteriosus constriction of 72 fetuses and reported that the greatest incidence of ductal constriction occurred at 31 weeks’ gestation. The earliest gestational age for ductal constriction was reported at 24.7 weeks’ gestation^(7)^. Therefore, it could be argued that the ductal effect of indomethacin is unpredictable for the foetus at any gestational age. Complete and irreversible closure of the ductus arteriosus can be observed even after a single dose of indomethacin, whereas ductus arteriosus can remain unaffected despite repeated doses. In our cases, constriction of the ductus arteriosus was seen only in the donor twin. In our opinion, this effect was observed only in the donor twins because of the relatively hypoxic conditions of donor foetuses. A selective constrictive effect of indomethacin on MC twins has been shown previously in some pathologically proven reports^(8)^. Furthermore, indomethacin can decrease fetal urine output and lead to deterioration in amniotic fluid levels in the donor foetus. Thus, it seems reasonable to use tocolytics other than indomethacin in TTTS. Just a few cases of hydrops and constriction of ductus arteriosus following indomethacin use have been described in the literature and none presented as early as 24 weeks’ gestation. As in our case, hydrops resolved after discontinuation of indomethacin^(9)^. Indomethacin use is associated with a risk of constriction of the ductus arteriosus at any gestational age; however, this condition and its effects can present in the absence of Doppler criteria. In case 1, the absence of Doppler criteria can be explained by the relatively early gestational age and severe tricuspid regurgitation. Tricuspid regurgitation allows the right ventricle to empty and may prevent turbulent flow in the ductus arteriosus. In conclusion, these are the first reported cases of foetal ductal constriction in complicated MC twin pregnancy presenting as early as 20 weeks’ of gestation. Therefore, we emphasize meticulous use of indomethacin in complicated MC twin pregnancies because the constrictive effect seems to be independent of gestational age. In the event of indomethacin use, Doppler indices of the ductus arteriosus should be closely monitored.
